# Active defense strategies for invasive plants may alter the distribution pattern of pests in the invaded area

**DOI:** 10.3389/fpls.2024.1428752

**Published:** 2024-07-11

**Authors:** Junjie Zhai, Bin Hou, Fangyu Hu, Guozhu Yu, Zhiqi Li, Evan C. Palmer-Young, Hui Xiang, Lei Gao

**Affiliations:** ^1^ Guangdong Provincial Key Laboratory of Biotechnology for Plant Development, School of Life Sciences, South China Normal University, Guangzhou, China; ^2^ United States Department of Agriculture- Agricultural Research Service (USDA-ARS) Bee Research Laboratory, Beltsville, MD, United States; ^3^ Guangdong Provincial Key Laboratory of Insect Developmental Biology and Applied Technology, Guangzhou Key Laboratory of Insect Development Regulation and Application Research, Institute of Insect Science and Technology, School of Life Sciences, South China Normal University, Guangzhou, China

**Keywords:** defense chemicals, *Sphagneticola trilobata*, *Mikania micrantha*, invasive plant, herbivory

## Abstract

**Introduction:**

In the invaded areas, it is believed that invasive species reduce their investment in defense due to the absence of natural enemies.

**Methods:**

By field investigation and a series of laboratory assays, This study explored the defense strategies of invasive plants.

**Results:**

Field investigation indicated that invasive plants have a antifeedant effect on herbivorous pests, and the distribution frequency of wormholes of native plants shows a peak at a distance of 2–3 m from the invasive species. The feeding preference experiment conducted with two generalist herbivorous insects (native insect *Spodoptera litura* and invasive insect *Spodoptera frugiperda*) showed that the invasive plants have a stronger antifeedant effect than native plants. By analyzing the content of secondary metabolites in the leaves of three invasive plants (*Sphagneticola trilobata*, *Mikania micrantha*, *Ipomoea cairica*) and three native plants (*Ipomoea nil*, *Paederia foetida*, *Polygonum chinense*), the leaves of invasive plants had higher concentrations of substances associated with defenses, including total phenols, flavonoids, jasmonic acid, tannin, H_2_O_2,_ and total antioxidant capacity (TAC), and lower soluble protein concentrations than native plants. After leaf damage, compared to native plants, the leaves of invasive plants showed an overall increase in substances associated with defense, except for soluble sugar.

**Discussion:**

These results suggest that invasive plants maintain active defense strategies in invaded areas, leading to changes in the distribution patterns of herbivorous insects in a manner that facilitates invasion.

## Introduction

Invasive plants have a serious impact on the ecology and environment of the invaded area. Many hypotheses have been advanced to explain the mechanisms of successful biological invaders (Joshi and Vrieling, 2005; Feng et al., 2009; Pal et al., 2020). These hypotheses have been discussed from different aspects: 1) the biological characteristics of the invasive species, such as the inherent superiority hypothesis (Sax et al., 2002), the novel weapon hypothesis (Callaway and Ridenour, 2004), the evolution of increased competitive ability (EICA), and the evolution of nitrogen allocation hypothesis (Feng et al., 2009); 2) the interaction between invasive species and native species, such as enemy release hypothesis (ERH) (Torchin and Mitchell, 2004); and 3) the invasibility of new habitats or environments, such as the empty niche hypothesis (Joshi and Vrieling, 2005) and disturbance hypothesis (Sax et al., 2002; Joshi and Vrieling, 2005). One of differences between invasive species and their origin places is the lack of threat from predators. ERH and EICA explains the relationship between the absence of predators and the successful invasion (Keane and Crawley, 2002; Heger and Jeschke, 2014). The ERH view is that the invasive species lack specialist natural enemies in the new habitats (Keane and Crawley, 2002; Liu and Stiling, 2006; Najberek et al., 2020). The competition between invasive species and native plants is greater than their intraspecific competition. Therefore, invasive species are less constrained by negative density, they have higher fitness and can allocate more resources to growth (Yu and Li, 2020), EICA emphasizes the balance between defense and growth allocation of invasive species, believing that invasive plants invest substances that were originally defensive against natural enemies into their growth, thereby enhancing their competitiveness (Blossey and Notzold, 1995; Joshi and Vrieling, 2005). Based on these viewpoints, the invasive species can allocate more resources on growth and reproduction in the new habitats and reduce defense investments. This could lead to the rapid spread of invasive species in the invaded area (Huang and Ding, 2016; Gonzalez-Teuber et al., 2017). However, the premise of this hypothesis is that an invasive plant should has one specialist enemy, and the population size is affected by the specialist enemy population, but, in fact most invasive plants do not have a single specialist enemy (Liu and Stiling, 2006). Studies have found that many of the natural enemies of invasive plants in their origin areas are specific predators, and the invasive plants still face the threat of generalist predators in the invasion site (Levine et al., 2004; Parker et al., 2006; Morrison, 2011).

Many studies have demonstrated that despite the lack of specific enemy, invasive plants did not reduce their investment in defense substances at new sites (Pysek and Richardson, 2010; Jack and Friesen, 2019). Compared to native plants, invasive plants suffer lower rates of damage caused by herbivorous insects (Manea et al., 2019), which suggests that invasive plants may have stronger defenses against generalist herbivores (Rotter and Holeski, 2018).

In the new environment, changes of invasive plant pests lead to evolutionary selection of invasive plant, which not only reduces invasive plant defense against specific predators, but also leads invasive plant to evolve towards enhancing the defense against generalist predators (Zhang et al., 2018). Based on the cost of different defense strategies invested in plants, some studies have proposed the shifting defense hypothesis (SDH). The hypothesis suggests that invasive plants do not exhibit complete changes from defense to growth and reproduction, but instead reduce their investment in defense against predators (reduced toxins) and increase their investment in defense against generalist insects (Joshi and Vrieling, 2005; Yi et al., 2024). If the production of defense substances is not more beneficial than the use of resources for growth and reproduction, plants should not produce these substances (Agrawal, 2011; Hinman et al., 2019). Therefore, the hypothesis that the invasion mechanism of invasive species is due to the energetic benefit realized from relaxation of chemical defenses (due to the lack of specialist enemies at the invasion site) remains speculative.

Many invasive plants can produce defense substances to prevent feeding by herbivorous pests (Pinzone et al., 2018). Defense substances produced by plants typically fall into two categories (Zhang et al., 2019). Some substances have a direct antifeedant effect on herbivorous insects, and these generally have toxic effects or reduce plant palatability (Zhang et al., 2019). Other substances are volatile and have a long-distance antifeedant effect on herbivorous insects (Zhang et al., 2019). In the wild environment, the number of herbivorous pests in a certain area should not be affected by invasive plants. If these herbivorous pests cannot feed on invasive plants, they will have to feed on native plants around the invasive plants.

Theoretically, a plant without other disturbance should be equally exposed to pests, so the distribution of herbivorous pests should be uniform. When invasive plants exist, due to their different defense strategies against pests, the invasive plants will change the distribution pattern of herbivorous insects. On this basis, the antifeedant effect of invasive plants can be tested by analyzing the damage pattern of native plants around invasive plants and then exploring the defense strategies of the invasive plants. Studies have documented antifeedant effects of invasive plants on herbivorous insects (Liu et al., 2020b; Dolma and Reddy, 2022). However, these studies mainly focused on the allelopathy of invasive plants and rarely involved the distribution patterns of native herbivorous insects that are impacted by invasive plants (Liu et al., 2020b). In Guangdong province, there are a large number of invasive plants present in abandoned farmlands. These invasive plants are usually *Mikania micrantha*, *Sphagneticola trilobata* and *Bidens alba* all of which belong to Asteraceae. Furthermore, we found that invasive plant leaves in abandoned farmland have fewer wormholes found for pests to feed on. On the contrary, in adjacent farmland crops, there are often more leaf wormholes that are eaten by pests. Therefore, this suggests that the presence of invasive plants may increase the degree to which herbivorous insects feed on native plants, and also suggests that invasive plants may have stronger defense capabilities than native plants.

Compared with original species of invasive plants, invasive plants may reduce defense investment due to the lack of natural enemies, but their defense should be positive compared with the native plants in the invasion site. To test whether invasive plants have an active defense strategy, we designed four sets of experiments to reveal the defense characteristics of invasive plants. We 1) investigated the foliar damage distribution pattern of native plants around invasive plants in the field to determine whether invasive plants can repel native insects; 2) conducted on the feeding preferences of herbivorous insects towards invasive and native plants under laboratory conditions; 3) compared the contents of secondary metabolites associated with plant defense in leaves of several invasive and related native plants; and 4) analyzed the contents of defense-related metabolites in the leaves of experimentally and naturally herbivore-damaged plants. Through these four aspects of study, we attempt to verify the hypothesis of the active defense of invasive plants and attempt to reveal the mechanisms of successful invasion.

## Methods

### Distribution patterns of herbivore damage of native plants

We selected four sample sites in Guangdong Province, including forest park, orchard, farmland and wasteland, and a total of 12 sample sites were randomly selected (**Supplementary Figure S1**; **Supplementary Table S1**). The invasive plant species are *M. micrantha*, *S. trilobata* and *B. alba*. These invasive species are commonly found in various disturbed habitats in Guangdong Province, such as in forest margins, farmland margins, orchards, forest parks, roadsides, pond edges, and abandoned farmland where crops are no longer planted. The impact of these invasive species on the local environment has attracted the attention of ecologists (Zhang et al., 2020; Liu et al., 2020a; Wang et al., 2020b; Yu et al., 2021). *M. micrantha* is a herbaceous vine with strong climbing ability. It can cover shrubs and lower trees, causing serious damage to native vegetation and fruit trees (Yu et al., 2021). *M. micrantha* has become one of the most invasive species in China (Zhou et al., 2005; Wang et al., 2008). *S. trilobata* is a creeping herb that can reproduce asexually through its stolons, forming a single dense population and resulting in a significant reduction of native plants (Gao et al., 2022). *B. alba*, known as an invasive weed that has a strong growth ability in withstand drought and unfertile soil, and it mainly propagates through seed dispersal (Wang et al., 2020b).

In the sampling sites, the growth of the invasive plants was not disrupted. The farmers did not spray pesticides and herbicides in orchards and farmland. In each site, we took an invasive plant as the center, investigated the leaf damage (leaf holes) found on the native plants around the invasive plant, measured the straight-line distance from the leaf damage to the invasive plant, and then counted the proportion of the number of damaged leaves at each distance to the total leaf damage number recorded (**Supplementary Figure S2**).

### Antifeedant effects of invasive plants on herbivorous insects

To further confirm the antifeedant effect of invasive plants on herbivorous insects, we conducted an insect antifeedant experiment in laboratory. We used three common invasive species, namely *S. trilobata* (Asteraceae), *M. micrantha* (Asteraceae), *Ipomoea cairica* (Convolvulaceae), and the following native species were selected as controls: *Sphagneticola calendulacea* (Asteraceae), *I. nil*, *Paederia foetida* (Rubiaceae), *Polygonum chinense* (Polygonaceae), and native crop species *Lactuca sativa* (Asteraceae), *Ipomoea batatas* and *Perilla frutescens* (Lamiaceae). In addition, a hybrid species of *S. trilobata* and the native species *S. calendulacea* was found in the South China Botanical Garden of the Chinese Academy of Sciences, Guangzhou, China. The adaptation of *S. trilobata* and the hybrid to water and cadmium have been studied (Zhang et al., 2020; Cai et al., 2021; Gao et al., 2022). The hybrid was selected to compare the antifeedant effects of invasive species on herbivorous pests. The antifeedant effect of invasive plants was tested using the feeding rates of the herbivorous insects *Spodoptera litura* and *S. frugiperda* larvae (fourth instar). *S. litura* has a worldwide distribution (Kamaraj et al., 2008) and is a native pest in China. *S. frugiperda* is an invasive insect in China (Goergen et al., 2016).

In the experiment, one insect was placed in the middle of a culture box and placed the same weight of fresh leaves of invasive species and native species on both sides of the box (**Supplementary Figure S2**). The leaves were weighted before the experiment and then placed the culture boxes in a ventilated light incubator at a constant temperature of 25°C for 24 h. After 24 h, the leaf leftover were photographed and dried to constant weight. The mass of leaves was calculated from each plant species eaten by the larvae (**Supplementary Figure S2**).

The detached leaves lost water continuously during the experiment. Before the antifeedant experiment, we randomly picked 15 pieces of leaves of each species under their natural state, weighed the fresh mass, and then put them into an oven. We set the temperature to 100–105°C for 10 min and then reduced the temperature to 75°C for heating the leaves to a constant weight. We then weighed the dry mass and calculated the average water content of the leaves of each plant species. Based on this water content, the dry mass of leaves of each was calculated. 5 repetitions, 3 invasive species, 7 native plants, and 2 insect species were tested. The total number of feed preference treat combination was 5 × 3 × 7 × 2 = 210.

### Induced defense chemicals

Insects may deposit oral secretions on leaves (Gaquerel et al., 2012; Giron et al., 2016). To exclude the effects caused by compounds deposited by the insects, we simulated insect feeding by drilling holes on the leaves to verify whether changes in the defense substances of invasive plants involve a general response to leaf damage. In addition, compared with insect feeding, mechanical drilling of leaves has the same damage degree to leaves, and the difference of defense substance content in leaves of different plants can be compared. The sampling site was on Changzhou Island, Guangzhou. The terrain of the site is flat, and the soil is uniform and lacks human interference. The habitats of all invasive plants and weeds in this area were similar.

In this study, three invasive species, *I. cairica*, *M. micrantha* and *S. trilobata* were selected for the experiment. Three native weeds, *I. nil*, *P. foetida* and *P. chinense*, were selected as corresponding controls. Both *I. cairica* and *I. nil* belong to the same family. *M. micrantha* and *P. foetida* are vine plants with similar morphological and ecological characteristics. *P. chinense* is a common native weed (Sun et al., 2019) that often appears in the same habitats as invasive plant *S. trilobata*. At each sampling site, twelve individuals of each species were sampled. Among these twelve plants, the leaves of six plants at the 4th–5th leaf position from the top of the branch were drilled with a coring tool, and two round holes with a diameter of 6 mm were drilled on both sides of the main leaf vein to simulate insect feeding on the leaves. The leaves of the other six control plants were not drilled. Before the experiment, we measured the dynamics of secondary metabolites over time after leaf damage in *M. micrantha* and *S. trilobata*, and the results showed that the secondary metabolites in leaves had significant peaks 24h after leaf damage (Hou, 2018; Zhai, 2021). Based on these findings, 24 h after leaf damage, we collected all the artificially drilling and control leaves, numbered the leaves, immersed them in liquid nitrogen, and returned them to the laboratory to measure their secondary metabolite content. To compare the difference between artificial drilling and insect feeding, we took six leaves with holes caused by insect feeding under natural conditions from the same sample sites, selected one leaf for each plant, numbered the leaves, immersed them in liquid nitrogen, and returned them to the laboratory for the determination of secondary metabolite content in the leaves.

The total phenol content was measured according to Folin Denis method (Ainsworth and Gillespie, 2007), and the flavonoid content was measured via aluminum salt (aluminum chloride) color spectrophotometry (Heimler et al., 2005). The condensed tannin content was measured using the vanillin hydrochloric acid method (Agullo and Rodriguez, 1995; Nakamura et al., 2003), and the soluble sugar content was measured via modified anthrone sulfuric acid colorimetry (Pernia et al., 2019). The soluble protein content was measured using a Bradford Kit (Shen et al., 2011; Wang et al., 2016). The total antioxidant capacity (TAC) was determined by the DPPH radical scavenging reaction method (Saha et al., 2008). The content of jasmonic acid and hydrogen peroxide was measured using an enzyme-linked immunosorbent assay (ELISA) kit (Shenzhen Zike Biological Company, Shenzhen, China) according to the manufacturer’s instructions.

### Data analysis

In the study of the distribution pattern of native herbivorous insects, we calculated the frequency of leaf holes on native plants at different distances from the invasive plants. The frequency of leaf holes = hole number at a certain distance/total hole number × 100%. We regressed the leaf hole frequency at different distances from invasive plants.

In the insect feeding preference experiment, only the mass and feeding rate of fresh leaves eaten by insects were examined. This study analyzed the differences of feeding rates of two insects using native plants minus invasive plants, and after Levene’s test for equality of variances, we used an independent samples t-test (two-tailed) for analyzing the significance of the feeding rates of the two insects on two species (native plants as the study group, invasive plants as the contrast group). In addition, a kind of non-parametric tests (rank-sum test) was used in cases where the data did not conform to a normal distribution, as this test is not limited by the distribution of the total samples. When we calculated the fresh leaf intake of insects, the water content of the plant leaves was determined before the feeding experiment. After drying and weighing the remaining leaves fed on by insects, the value was converted into fresh weight according to the leaf water content, and then, the feeding intake of the insects was calculated by the difference of leaf fresh mass before and after feeding. The calculation formula was as follows:


$$\begin{array}{c} \text{Lea water content of plant species }=\\ \left(\text{Fresh weight Dry weight}\right)\text{/Fresh weight} \end{array}$$

$$\begin{array}{c} \text{Feed intake of fresh leaves = }\\ \left(\text{Fresh weight - Dry weight after feeding}\right)/\left(1 –Water content\right) \end{array}$$

Feeding rate = Feed intake/Weight of fresh leaves ×100%

In order to explore the response of secondary metabolite content in invasive plant leaves after mechanical damage or herbivorous feeding, we used one-way analysis to compare the differences in secondary metabolites in the leaves of invasive species and native species before damage, after mechanical damage, and after insect damage. The differences between invasive plant and native plant were compared by a least significant difference test (LSD). The hypothesis test level of analysis of variance α = 0.05. All statistical data analyses were performed with SPSS^®^V22.0, and graphs were created with Origin 2018.

## Results

### Effects of invasive species on the frequency distribution of leaf holes in native plants

**Figure 1:**
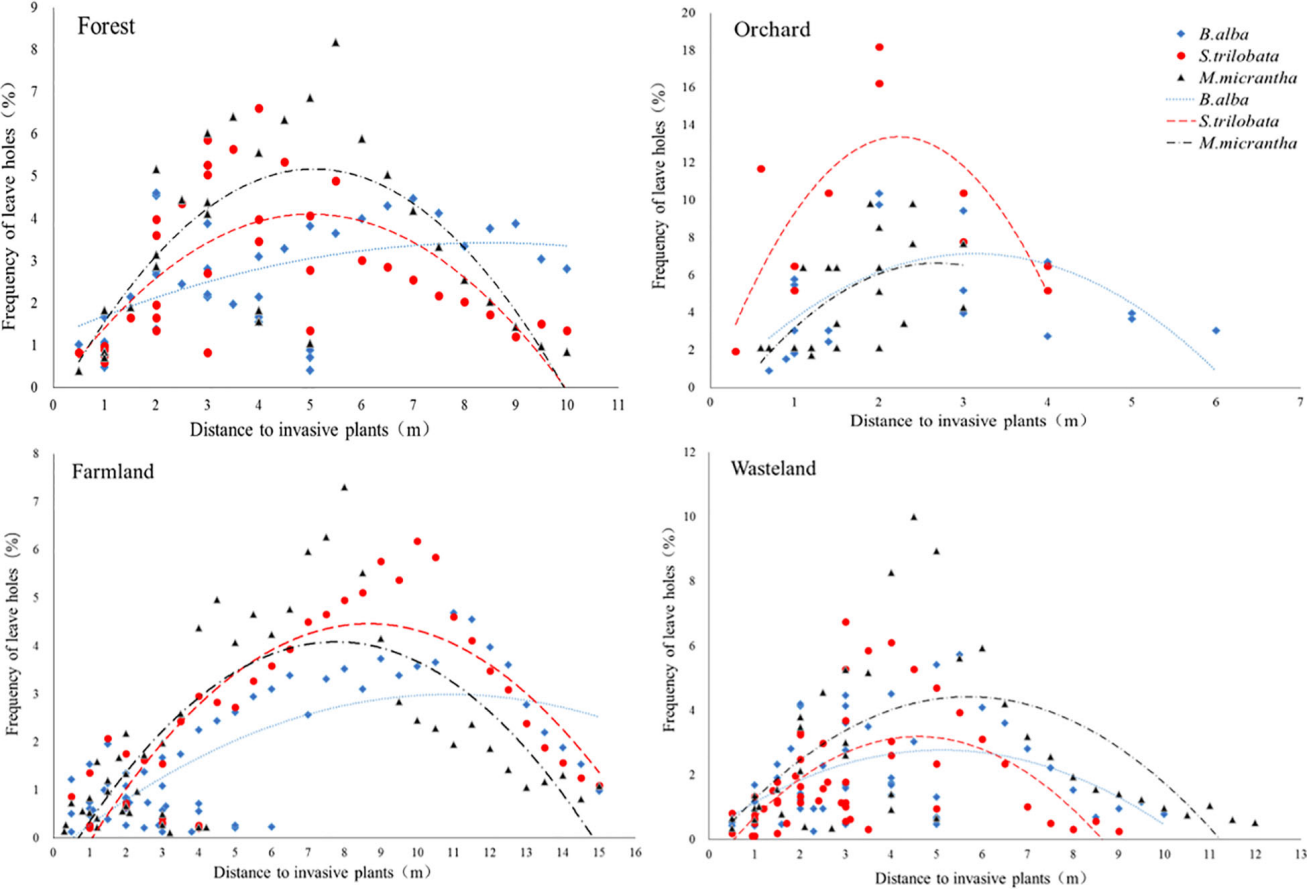
Frequency of leaf holes on native plants at different distances from the invasive plants: *B. alba*, *S. trilobata* and *M. micrantha*—in four kinds of habitat—forest, orchard, farmland and wasteland—showing the effect of invasive plants on the native insect feeding distribution pattern. The curve illustrates the quadratic regression of the distribution frequency of herbivore damage at different distances from invasive plants.

The number of holes on native plant leaves had parabolic characteristics regressed with the distance to invasive plants, and there was a peak in the distribution of leaf holes at the distance from invasive plants (**Figure 1**). The regression curves of *B. alba* were the flattest among the three invasive species in the four habitat types. *S. trilobata* and *M. micrantha* were similar and had higher peaks of leaf hole distribution than that of *B. alba*. However, the peak values of the three invasive species varied in the sample sites. *S. trilobata* had the greatest impact on the distribution of leaf holes of native plants in orchards. In orchards, there was a high frequency of leaf holes 2–3 m away from *S. trilobata* (**Table 1**). More leaf holes in plants indicate more pest distribution. So, the distribution of herbivorous pests reaches its peak at a distance of 2-3 meters from *S. trilobata*, which indicated that *S. trilobata* significantly changed the distribution pattern of herbivorous pests in orchards. In addition, the distance of leaf hole peaks from different invasive plants was different. Among the three invasive species, the peak value of leaf hole distribution was nearest to *S. trilobata*. The peak distance was the longest in farmland and the shortest in orchards. Therefore, the peak distance of holes showed that in orchards, the impact of invasive species on herbivorous pests was weaker, while in relatively open and single crop farmland, the impact distance of invasive species was greater (**Table 1**).

**Table 1:** Binomial regression analysis of frequencies of leaf hole distribution observed on native plants at different distances from three invasive plants in four kinds of habitats.

Habitat	Invasive plant	Total holes	df1	df2	R^2^	Peak of holes (m)	*p*	F
Forest	*B*. *alba*	1672	2	35	0.208	8.45	0.017	4.60
	*S. trilobata*	1328	2	33	0.451	5.01	0	13.54
	*M. micrantha*	1528	2	26	0.505	5.07	0	13.29
Orchard	*B*. *alba*	328	2	17	0.336	3.13	0.031	4.29
	*S. trilobata*	154	2	8	0.536	2.23	0.047	4.61
	*M. micrantha*	234	2	17	0.324	2.69	0.036	4.07
Farmland	*B*. *alba*	3770	2	63	0.564	10.89	0	40.72
	*S. trilobata*	3814	2	35	0.69	8.66	0	38.02
	*M. micrantha*	3585	2	46	0.569	7.77	0	30.263
Wasteland	*B*. *alba*	1881	2	47	0.207	5.11	0	6.11
	*S. trilobata*	1570	2	49	0.352	4.59	0	13.34
	*M. micrantha*	2259	2	35	0.357	5.69	0	9.63

### Antifeedant effects of three invasive species on two larvae of Noctuidae

**Figure 2:**
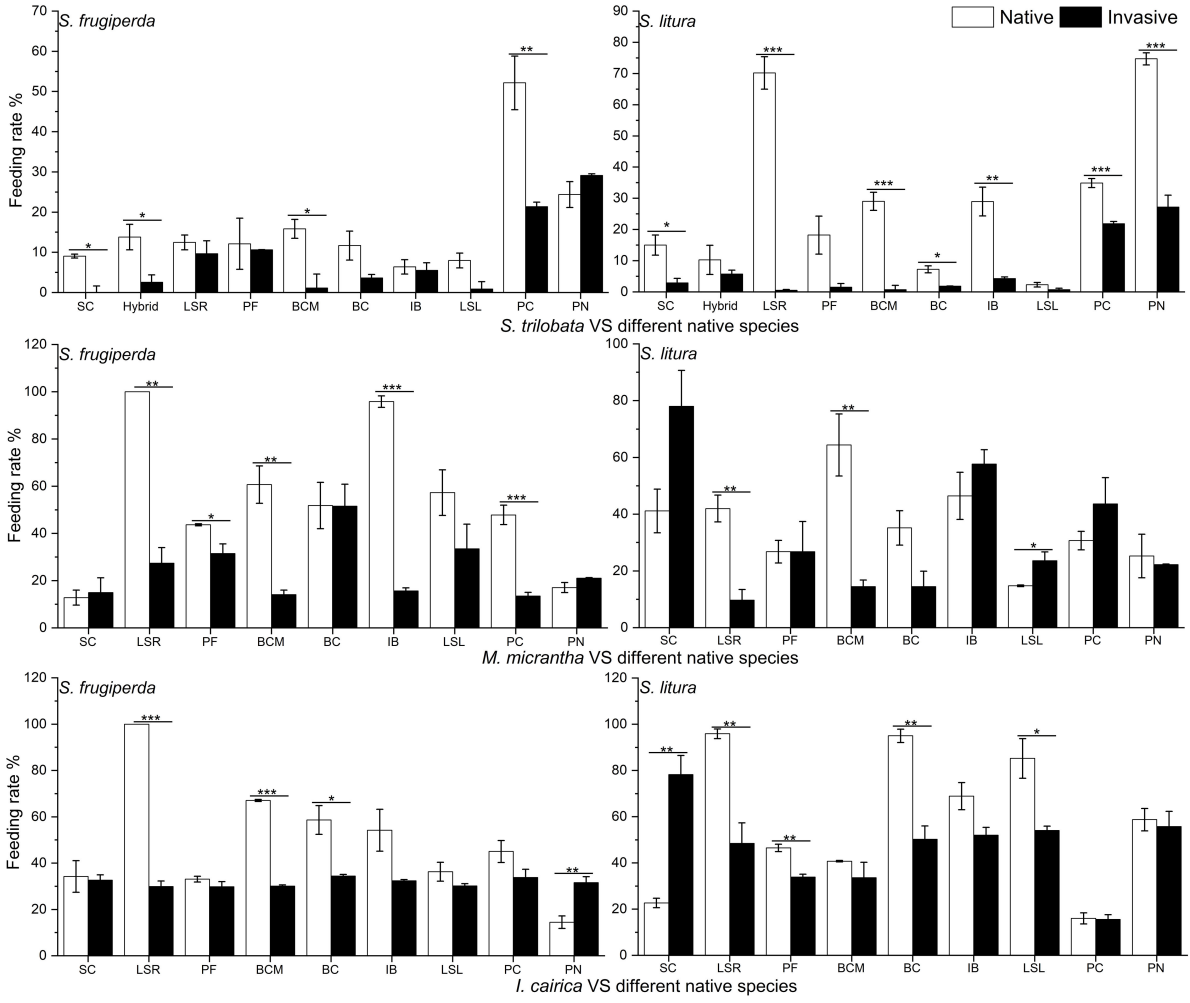
Comparison of feeding rates of two kinds of insects (native insect: *S. litura* and invasive insect: *S. frugiperda*) on the invasive plants *S. trilobata*, *M. micrantha*, *I. cairica* and the native plants: *S. calendulacea* (SC), *P. chinensis* (PC), *P. scandens* (PS), *I. nil* (IN), *B. chinensis* (BCM), *Lactuca sativa* R. (LSR), *Lactuca sativa* L. (LSL), *P. frutescens* (PF), *B. campestris* (BC), *I. batatas* (IB). The mean ± SE are shown. Asterisk (*) indicate significant differences between the feeding rates invasive and native species determined using independent samples *t*-test (two-tailed) of feeding rate between invasive and native species (*p*<0.05).

Insect feeding experiments showed that when two kinds of insects (*S. litura* or *S. frugiperda*) were placed between invasive and native plants, insects fed more on native plant leaves (**Supplementary Figure S2**). We calculated the feeding intake of the two insects feeding on the leaves of invasive species and native species. The difference value of feeding intake by native plants minus invasive plants were mostly positive, so the proportion of insects selecting on native plants was larger than that on invasive plants (**Table 2**). Therefore, the feeding rates of insects on native plants were mostly higher than that on invasive plants (**Figure 2**). The results showed that invasive plants can have antifeedant effects on herbivorous insects.

**Table 2:** Difference value in feeding intakes through native plants minusing invasive plants (mean difference ± SE), and independent samples t-test (two-tailed) of the feeding intakes on two species (native plants as study group, invasive plants as a contrast group), df = 4 (after Levene’s test for equality of variances, when the variance is homogeneous, df = 4, and when the variance does not meet the homogeneity, df ≠4).

Invasive species	Native species	Native insect: *S. litura*			Invasive insect: *S. frugiperda*		
		Difference	t	*p*	Difference	t	*p*
*S. trilobata*	*S. calendulaceae*	.12 ± .012	3.44	.026	.13 ± .038	7.64	.002
	Hybrid	.09 ± .019	.94	.401	.11 ± .072	3.06	.038
	*P. chinensis*	.13 ± .057	8.00	.001	.31 ± .020	4.56	.01
	*I. nil*	.48 ± .028	10.98	0	−.05 ± .037	−1.46	.278
	*Lactuca sativa* R.	.70 ± .05	13.36	0	.03 ± .037	.75	.494
	*P. frutescens*	.17 ± .121	2.70	.106	.02 ± .064	.24	.833
	*B. chinensis*	.03 ± .044	8.87	.001	.15 ± .042	3.50	.025
	*B. campestris*	.28 ± .049	4.92	.039	.08 ± .037	2.19	.094
	*I. batatas*	.27 ± .037	5.23	.006	.01 ± .026	.33	.756
	*Lactuca sativa L.*	.02 ± .025	1.89	.132	.07 ± .004	2.74	.052
	Proportion of choosing native plants	100%>0			90%>0		
*M. micrantha*	*S. calendulaceae*	−.12 ± .329	−2.48	.068	−.02 ± .092	−.30	.78
	*P. chinensis*	−.13 ± .103	−1.33	.256	.34 ± .042	7.78	.001
	*I. nil*	.02 ± .079	.39	.714	.06 ± .102	1.851	.138
	*Lactuca sativa R.*	.27 ± .100	−5.32	.006	.73 ± .066	11.02	.008
	*P. frutescens*	.00 ± .138	.00	.997	.16 ± .221	2.93	.043
	*B. chinensis*	.13 ± .259	.46	.011	.37 ± .121	5.72	.005
	*B. campestris*	.21 ± .107	2.54	.064	.17 ± .289	.02	.985
	*I. batatas*	−.11 ± .051	−1.15	.315	.66 ± .129	29.27	0
	*Lactuca sativa L.*	−.37 ± .223	−2.81	.048	.24 ± .135	1.68	.169
	Proportion of choosing native plants	56%>0			88.9%>0		
*I. cairica*	*S. calendulaceae*	−.60 ± .075	−6.535	.003	.02 ± .064	.22	.834
	*P. chinensis*	.04 ± .059	.15	.886	.11 ± .040	1.91	.129
	*I. nil*	.00 ± .308	.36	.735	−.17 ± .040	−4.61	.01
	*Lactuca sativa R.*	.47 ± .107	5.18	.007	.39 ± .288	28.89	0
	*P. frutescens*	.13 ± .022	6.27	.003	−.07 ± .083	1.27	.272
	*B. chinensis*	−.01 ± .109	1.067	.346	.22 ± .154	53.79	0
	*B. campestris*	.25 ± .250	6.905	.002	.09 ± .162	3.86	.018
	*I. batatas*	.01 ± .194	2.497	.067	.22 ± .092	2.41	.137
	*Lactuca sativa L.*	.22 ± .129	3.57	.023	.27 ± .208	1.47	.215
	Proportion of choosing native plants	77.8%>0			77.8%>0		

A positive value of t indicates that the feeding intake on native species is higher than that on invasive species. For decimals less than 1, we omit the 0 before the decimal point. Hybrid refers to a hybrid between the invasive plant *S. trilobata* and the native plant *S. calendulaceae*. Different colors represent native weeds and crops (native weeds: green, crops: yellow). The proportion of native plants selected by insects is obtained by calculating the proportion of every mean difference greater than zero in all insect feeding preference experiments.

The insect feeding preference experiment showed that the intensity of antifeedant effect of the three invasive species on insects was variable (**Table 2**; **Supplementary Figure 2**). For the invasive species *S. trilabata*, significance was found in 11 out of 20 contrasts to native plants (*p*<0.05). The feeding rates of the two insects were significantly higher on native plants (21.35%) than on *S. trilobata* (7.6%). Compared to *S. trilobata*, the antifeedant effect of the invasive *M. micrantha* was weaker; the feeding rates of the two insects on native plants were 42.5%, and the feeding rates on the invasive plant *M. micrantha* were 33.05%. Among the three invasive species, the antifeedant effect of *I. cairica* was the weakest, with the feeding rates of the two insects on native plants being 49.3% and on *I. cairica* being 40.0% (**Table 3**; **Supplementary Figure 1**). Despite its antifeedant effect, *I. cairica* was still less preferred than some native cultivated plants (*L. sativa*, *P. frutescens*) and the native weed *S. calendulaceae* (**Table 2**; **Supplementary Figure 2**).

**Table 3:** Feeding rates of two insects feed on the leaves of three invasive plants and native plants, mean ± SE.

Invasive sp.	Native insect *S. litura*		Invasive insect *S. frugiperda*	
	invasive sp.	native spp.	invasive sp.	native spp.
*S. trilobata*	7.1 ± 1.29	27.1 ± 3.14	8.1 ± 1.87	15.6 ± 0.03
*M. micrantha*	38.8 ± 5.38	33.6 ± 5.75	27.3 ± 5.12	51.4 ± 4.85
*I. cairica*	48.6 ± 5.07	53.3 ± 3.31	31.4 ± 1.65	45.3 ± 3.69

The antifeedant effects of the invasive species differed between the two insect species. As evidenced by the experiments with *S. trilobata*, the native insect *S. litura* (27.1%) has a higher preference for feeding on native plants than invasive insect (15.6%) (**Table 3**). The feeding rate of invasive insect *S. trilobata* on the other two invasive plants is lower than that of native insects, but the feeding rate of invasive insects on native plants is greater than that on invasive plants, indicating that invasive insects also have a feeding preference (**Table 3**).

### Responses of three invasive plant defense-related substances to leaf damage

**Figure 3:**
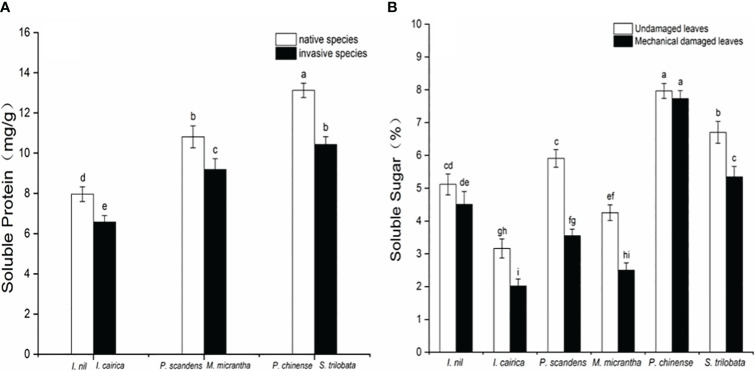
Soluble protein and soluble sugar content in the leaves of three invasive plants (*I. cairica*, *M. micrantha*, *S. trilobata*) and three native plants (*I. nil*, *P. foetida*, *P. chinense*) in the field conditions, **(A)** soluble protein content of native and invasive species; **(B)** soluble sugar content of undamaged and mechanically damaged leaves, showing the mean ± SE. Different lowercase letters indicate statistically significant differences among different invasive and native species (*p*<0.05) obtained via one-way analysis of variance with a *post-hoc* Tukey test.

We measured the soluble protein content in the leaves of three invasive species and three native species under field conditions. The soluble protein contents of the three invasive species were all significantly lower than those of the native species (**Figure 3A**). This showed that, before being damaged, the invasive species had a reduced content of soluble protein in leaves available for growth input. The content of soluble sugar in the leaves damaged by mechanical drilling in invasive species was significantly lower than that in the undamaged control leaves (**Figure 3B**). This suggests that, after being damaged, the invasive species reduced the content of soluble sugar used for growth and increased the levels of defensive chemicals.

**Figure 4:**
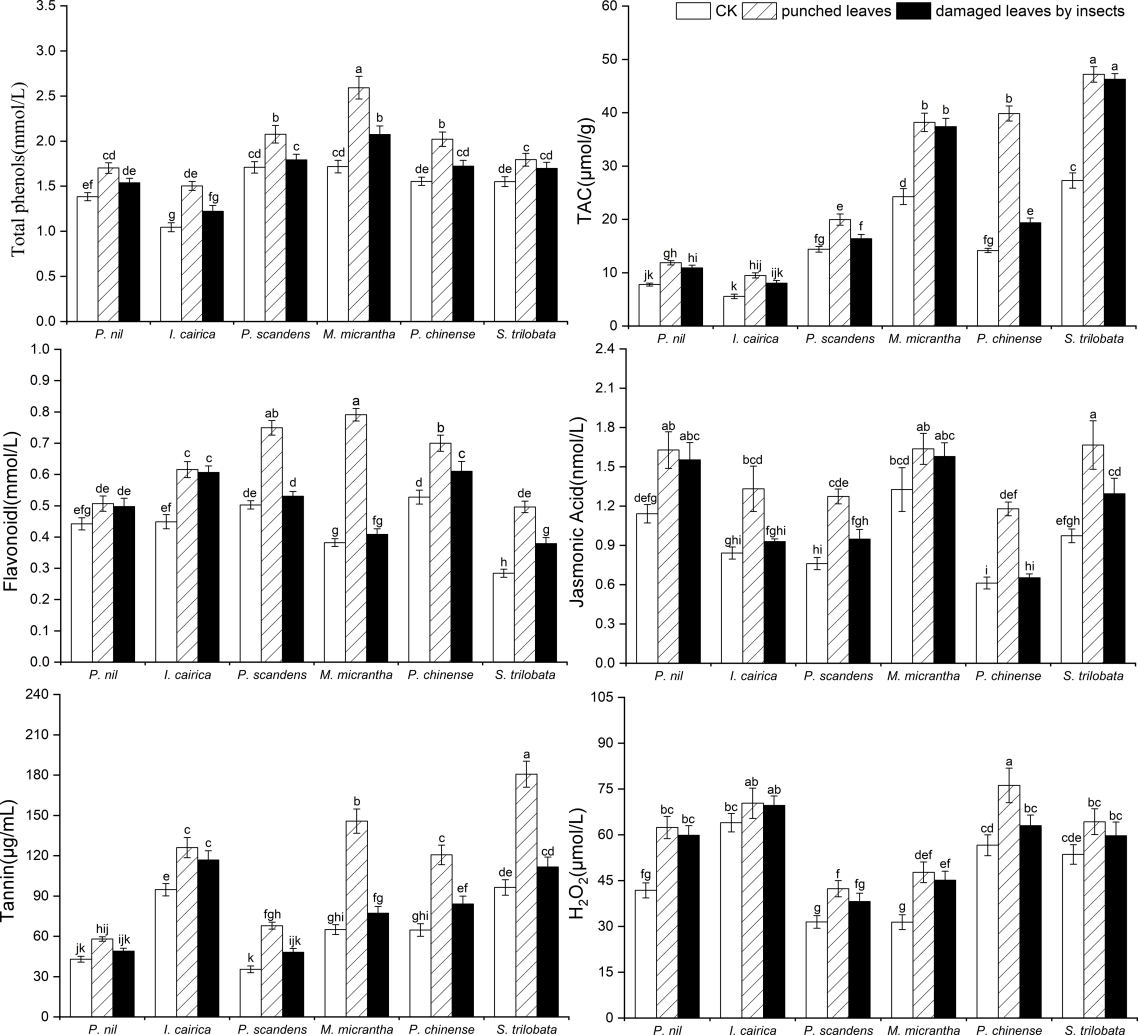
Phenolic compound contents (total phenols, flavonoids, and tannin) and comparison of the metabolite contents (jasmonic acid and H_2_O_2_) and TAC in the leaves of native species and invasive species (in bold font) (*I. nil and I. cairica, P. foetida and M. micrantha, P. chinense and S. trilobata*) before and after undergoing mechanical and insect damage in field conditions. The mean ± SE are shown. Different lowercase and capital letters indicate statistically significant differences (*p*< 0.05) among different invasive and native species based on one-way analysis of variance with a *post-hoc* Tukey test.

We measured the contents of antioxidant substances and signal molecules in the leaves of three invasive species and three native species under wild conditions (**Table 4**). The contents of total phenols, flavonoids, tannins, jasmonic acid, hydrogen peroxide and TAC in the leaves of invasive species were higher than those of the native species. For one invasive species, at least one index was higher than the native species (**Figure 4**). Therefore, these indexes were variable in the three invasive species. For example, the total phenol, jasmonic acid and TAC of the invasive species *I. cairica* leaves were lower than that of native species *I. nil*, but other indexes, such as the tannin and hydrogen peroxide content of *I. cairica* leaves, were significantly higher than those of *I. nil* (*p*< 0.01) (**Figure 4**); the flavonoid content of the invasive species *M. micrantha* was lower than that of the native species *P. foetida*, but other material contents were higher than those of *P. foetida* (**Figure 4**). In contrast, the flavonoid contents and hydrogen peroxide content of the invasive species *S. trilobata* were lower than those of the native species *P. chinense*, but its TAC and tannin content were significantly higher than those of *P. chinense* (*p*< 0.01) (**Figure 4**).

**Table 4:** Variance analysis (one-way analysis of variance) of metabolites contents of leaves between invasive species (*I. cairica*, *M. micrantha*, *S. trilobata*) and native species (*I. nil*, *P. foetida*, *P. chinense*) before and after being mechanical or insect damage in field conditions.

Dependent variable		Undamaged	Mechanical damage	Herbivory	Change ratio under mechanical damage	Change ratio under insect damage
Soluble protein	F	18.12				
	*p*	0.00				
Soluble sugar	F	28.34	35.82			
	*p*	0.00	0.00			
Total phenols	F	3.93	0.12	0.08	6.57	3.16
	*p*	0.05	0.73	0.78	0.01	0.08
Flavonoids	F	52.21	0.48	14.00	54.41	9.54
	*p*	0.00	0.49	0.00	0.00	0.00
TAC	F	28.55	11.12	60.33	6.74	21.36
	*p*	0.00	0.00	0.00	0.01	0.00
Tannin	F	93.63	102.26	68.06	1.32	1.45
	*p*	0.00	0.00	0.00	0.25	0.23
Jasmonic acid	F	0.07	2.89	6.08	2.04	0.05
	*p*	0.79	0.09	0.01	0.15	0.83
H_2_O_2_	F	4.79	0.02	1.04	2.56	0.33
	*p*	0.03	0.90	0.31	0.11	0.57

For soluble protein, only the undamaged leaves of invasive and native species were measured, and for soluble sugar, only the undamaged and mechanically damaged leaves of invasive and native species were measured. The change ratio is the difference value in metabolites content before and after the leaves underwent mechanical or insect damage (herbivory).

After leaf mechanical damage, the secondary metabolite contents in plant leaves increased (**Figure 4**); further, the change ratio of total phenols, flavonoids and TAC content was significantly higher in invasive species than in native species (*p*< 0.05) (**Table 4**). The response of metabolites in different invasive species to leaf mechanical damage also differed. The increase of flavonoid and tannin in the leaves of *M. micrantha* after mechanical damage was significantly greater than that of the other two invasive species (*p*< 0.05) (**Figure 4**). Among the invasive plant species, the increase of TAC and jasmonic acid was not greater in *M. micrantha*. The increase of these two indicators in *S. trilobata* was greater than the other two invasive species (**Figure 4**). Compared with *M. micrantha* and *S. trilobata*, the increase of leaf metabolites in *I. cairica* was relatively lower, except for the level of total phenols.

Analysis of the metabolite contents within damaged leaves of species in the field showed that the indexes of insect damaged leaves of invasive species were similar or lower than those of mechanically damaged leaves of the same species (**Table 4**, **Figure 4**). However, the indexes were significantly higher than those of undamaged controls (i.e., complete leaves) (**Figure 4**). This showed that the plant leaves can significantly increase their contents of metabolites after insect damage and can also maintain these metabolites at a high level. There were differences in the responses of different metabolites among the invasive species. In *I. cairica*, the contents of total phenol and jasmonic acid in the leaves damaged by insects were lower than those in the leaves damaged by mechanical drilling, and the contents of other metabolites were not significantly different from those in mechanically damaged leaves. However, in *M. micrantha*, the contents of total phenol, flavonoid and tannin in insect damaged leaves were significantly lower than those in mechanically damaged leaves, indicating that these metabolites increased first and then decreased to a constant level after damage. Unlike *M. micrantha*, in *S. trilobata*, the total phenols in the damaged leaves did not change significantly. This showed that the strategies of different invasive species to insect damage are different. Some substances are maintained at a high level after leaf damage, while other substances will return to a normal level after leaf damage.

## Discussion

### Effects of invasive plants on feeding patterns of native herbivorous pests

By investigating the damages of plants, the distribution of herbivorous pests can be reflected. In an open environment without human interference, the distribution frequency of leaves that are fed by herbivorous pests should be uniform for a certain plant. If invasive plants lack defensive substances, the distribution frequency of leaf damage on native plant should be uniform at different distances. After the distribution peak value of leaf holes, comparing with the number of peak value, the number of leaf holes in the leaves of native plants tended to decrease and remained at a stable level (**Figure 1**), indicating that the distribution of herbivorous pests is less affected when far away from invasive plants. Therefore, the number of leaf holes in the leaves of native plants at a distance could be used as a control to compare the influence of invasive plants on the distribution of leaf holes. In this study, the distribution frequency of leaf damage showed a peak at a distance of 2–3 m, indicating that the presence of invasive plants changed the original leaf damage distribution pattern. Therefore, this indirectly tested the possibility that invasive plants changed the feeding behavior of herbivorous insects. At a certain distance from three invasive species, the distribution frequency of leaf damage increased. This suggested that the herbivorous insects that may have been distributed among the invasive plants chose to eat native plants at a certain distance from the invasive plants. This also indicated an antifeedant effect of the invasive plants on herbivorous insects. Most previous studies on the antifeedant effect of plants on herbivorous insects were based on metabolites produced by plants after being fed upon by the insects, resulting in decreased palatability. For example, the feeding of *S. litura* on soybean leaves can induce the synthesis of flavonoids such as isoflavone glycosides and formononetin (Murakami et al., 2014). *Helicoverpa armigera* larval feeding on cotton can induce the synthesis of tannins, gossypol and other substances (Dixit et al., 2020), but there are only few studies on antifeedant effects of plants on the distribution pattern of insects. The altered insect distribution pattern confirmed the antifeedant effect of the invasive plants. The existence of invasive plants increases the threat of pests to nearby native plants, weakens the competitiveness of the surrounding native plants and promotes the further expansion of the invasive plants.

Study of the three invasive species showed that the effects of different invasive species on herbivorous insects can differ. From the peak height and steepness of binomial regression, the higher or steeper the peak value, the greater the influence of invasive plants on the distribution pattern of herbivorous pests. *S. trilobata* significantly changed the distribution pattern of herbivorous pests in orchards, while *M. micrantha* significantly changed the distribution pattern of herbivorous pests in forests and wastelands. This is also consistent with the vegetation types these invasive species often invade. *S. trilobata* can be distributed in the humid environments under the orchard canopy (Xie et al., 2010; Qi et al., 2020), while *M. micrantha* is often distributed on forest edges, forest gaps and wastelands (Banerjee et al., 2017). The antifeedant effect of *M. micrantha* on forest insects may also facilitate its successful invasion into the forest. Therefore, the differences in the antifeedant effect of different invasive plants against herbivorous pests also confirmed that the defense strategy is not the same among different invasive species.

### Antifeedant effects of invasive plants on insects

The distribution pattern of leaf holes in the field indicated an antifeedant effect of invasive plants on insects, but it was unclear what kind of herbivorous insects were being affected. Therefore, the herbivorous insect feeding preference experiments were used to study the antifeedant effect of invasive plants on different kinds of insects. The different feeding rates of the two insects on the leaves of invasive and native plants further confirmed that the invasive plants had an obvious antifeedant effect on herbivorous insects. When the leaves of invasive plant and native plant were placed together, the insects, whether native or invasive, choose to feed more native plants, it is further confirmed that under field conditions, the presence of invasive plants increases the threat of insect herbivory to native plants. In addition, the feeding experiments of *S. litura* and *S. frugiperda* showed that the three invasive species had different antifeedant effects on these two species. Among the three invasive plants, the antifeedant effect of *I. cairica* was the weakest, but the other two species (Asteraceae) had relatively strong antifeedant effects. Many species of Asteraceae produce strong volatile odors that can affect insect feeding behavior (Hori et al., 2006; Kleine and Muller, 2011). This is important evidence that species of Asteraceae have strong chemical defense mechanisms. In our experiment, we demonstrated that the antifeedant effect of invasive plants (such as *S. trilobata*) on a native insect (*S. litura*) was significantly greater than the effect on an invasive insect (*S. frugiperda*). This suggests that *S. litura* will increase its feeding on native plants due to the antifeedant effect of invasive plant species (such as *S. trilobata*). *S. frugiperda* is an invasive insect (Cock et al., 2017), and it origins to the Americas and was first discovered in Yunnan, China in 2019 (Zhao et al., 2019). *S. frugiperda* has now invaded most parts of China and brought a great threat to China’s crops and vegetation due to its wide feeding habits and rapid reproduction (Wan et al., 2021). In this experiment, although the feeding preference of the invasive insect is lower than that of the native insect (**Table 2**), the feeding rate of the invasive insect on native plants is higher than that of invasive plants (**Table 3**). This indicates that although the feeding preference of the invasive insect is weak, it will consume a large amount of native plants when invasive plants exist. It was also showed that the antifeedant effect of the invasive plant on *S. frugiperda* was weaker than that on the native insect *S. litura*. From the perspective of insect feeding, invasive insect *S. frugiperda* fed more on invasive plants than the native insect, which indicates that the invasive insect has strong invasiveness. For invasive plants, the defense strategies generated by invasive plants are mainly focused on native herbivorous insects rather than on invasive insects. This finding also shows that native insects are the main threat to invasive plants in invaded areas. In the process of invasion, native herbivorous insects constitute the main threat faced by invasive plants, while invasive insects are more uncertain to invasive plants. Therefore, the defenses of invasive plants are more effective against native insects. Invasive plants retain strong defense capabilities, which have been commonly described in recent studies. A growing body of research results indicate that invasive plants have a positive broad-spectrum defense ability against herbivorous pests in invaded areas (Parker et al., 2006; Wan et al., 2018), and invasive plants generally have strong allelopathic effects and special flavors (Zhang et al., 2019; Qi et al., 2020; Kato-Noguchi and Kato, 2023) that make them less susceptible to local herbivorous pests. This also indicates that invasive plants have an antifeedant effect on herbivorous pests.

### Response of invasive plants to leaf damage in defense substances

The plant defenses are mainly divided into two types: constitutive defense and inducible defense. Constructive defense is the inherent defense behavior of plants before being preyed upon by herbivorous insects, such as plant trichome, thorns, etc (Sanson et al., 2001; Ibanez et al., 2013). After being fed by herbivorous insects, many plants undergo inducible defense, synthesizing various defense compounds (such as total phenols, flavonoids, tannins, and other phenolic substances (Pass et al., 1998; Moore et al., 2004; Ito and Sakai, 2009; Wang et al., 2012). Plants can also synthesize protease inhibitors to inhibit the activity of protease in insect intestines, affecting their digestive ability and even their growth and development, thereby reducing their feeding behavior. When plants are subjected to pest stress, their signaling pathways are also activated, and the contents of signaling molecules such as jasmonic acid, salicylic acid, ethylene, and hydrogen peroxide will also change accordingly (Zhang et al., 2011; Wang et al., 2012; Becerra, 2015). In this study, the levels of secondary metabolites in the leaves of invasive plants further confirmed that invasive plants have more inducible defense strategies than native plants. Invasive plants have a strong antifeedant effect on pests in the field, which is consistent with their high contents of defense-related substances. The secondary metabolite contents of the different invasive plants were variable, which indicated that different species of invasive plants can have different chemical defense strategies. It may be related to their environment or the species of herbivorous insects attacking the plant. For those leaves that were not damaged, the contents of secondary metabolites in the three invasive plants were not lower than the corresponding native plants, indicating that the invasive plants maintained active defense strategies despite the apparent lack of specialist enemies in their invaded area. In the hypothesis of enemy release on invasive plants, it was suggested that invasive plants reduce their production of defense substances due to the absence of specialist enemies. The resources formerly devoted to defense are then allocated to growth and reproduction (Joshi and Vrieling, 2005). The premise of this hypothesis is that the production of chemical defense substances by plants consumes more energy than plant growth and reproduction (Neilson et al., 2013). Therefore, from the perspective of maximizing resource utilization, plants will allocate more resources to growth and reproduction in the absence of specialist enemies. However, our research confirmed that aggressive chemical defense of invasive plants in the invasion area may bring greater benefits, because the invasive plants may be threatened by generalist insects. Some invasive species have specialist enemies in their region of origin. For example, *Eichhornia crassipes* has a specialist enemy, a type of weevil (*Neochetina eichhorniae*), in its origin area in South America (Wilson et al., 2006), but this weevil is absent in China (Zhang et al., 2021). Therefore, invasive plants allocate energy for growth and reproduction, which can be explained by the EICA hypothesis. However, this does not explain well why invasive species still lack generalist pest infestations. For invasive plants, retaining or increasing chemical defenses may be more beneficial to invasion and range expansion than increasing the investment in growth and reproduction.

According to the ERH, invasive plants should carry out more compensatory growth after being damaged by herbivorous insects. This hypothesis holds that the allocation of resources to growth and reproduction may be the best strategy for plants (Joshi and Vrieling, 2005). However, our analysis of chemical defense substances in the leaves of invasive plants after mechanical damage indicated that the defense substances increased significantly. This showed that, for invasive plants, increasing the level of chemical defense substances should be the best allocation of resources after being damaged. The increase of chemical defense substances would bring greater benefits to reproduction and population expansion than would compensatory growth. Cipollini et al. studied the North American invasive plant *Alliaria petiolata* and native plants and found that compared with native plants, the invasive plants had higher contents of secondary metabolites related to defense. Aside from the tendency for invasive populations to have reduced constitutive glucosinolate levels coupled with increased inducibility, little support for the predictions of EICA was evident in the chemical defenses that studied (Cipollini et al., 2005). By analyzing the content of secondary metabolites before and after leaf damage, this study confirmed that invasive plants not only retain high defense characteristics before leaf damage, but also take active defense strategies after leaf damage.

Soluble sugar and soluble protein attract insects, which tend to choose plants with richer nutrients as their main food source. Plants with higher soluble protein content can enable herbivorous insects to obtain a higher survival rate, enhanced growth and increased reproduction (Wang et al., 2020a). Plant soluble sugar can directly stimulate the feeding behavior of insects and is an important nutrient and direct energy source for animals (Cheng et al., 2013). In our study, the soluble protein content in the leaves of invasive plants was lower than in the leaves of native plants, indicating that they have reduced palatability. This indicated that invasive plants do not increase their input of protein in growth and reproduction. When the leaves of invasive plants were mechanically damaged, the content of soluble sugar decreased significantly, and this further indicated that invasive plants quickly transfer energy to the production of defense-related substances after damage. Under pest stress, invasive plants increase their defense investment rather than increasing compensation investment for vegetative growth.

We also found that the contents and inducibility of secondary metabolites varied among different invasive plants. This indicated that invasive plants differ in their defense strategies, and different secondary metabolites may have different defense capabilities. Phenolic compounds are mainly total phenols, and common phenolic compounds include flavonoids and tannins. The contents of phenolic compounds secreted by plants can be regarded as a primary indicator of the strength of plant chemical defense. Although the contents of total phenols and flavonoids of the invasive plants were similar to those of native plants before damage, the contents of total phenols or flavonoids in the leaves of invasive plants (*I. cairica*, *S. trilobata* and *M. micrantha*) increased significantly after mechanical damage. In contrast, the tannin content in the leaves of invasive plants was significantly higher than that in the leaves of native plants before damage, which showed that the invasive plants maintained a high tannin content when they were not damaged by herbivorous insects. The EICA hypothesis suggests that compared to origin species, invasive species reduce their distribution of defense materials due to the lack of specialist enemies. However, the defense substance content analysis conducted in the present study revealed that the defense substances of invasive plants at the invasion site were retained at levels that matched or exceeded those of native plants, and the defense investment of the invasive species was not reduced due to the possible lack of natural enemies. Compared to the EICA hypothesis, the successful invasion of invasive plants may be attributed to the “ecological filtering” effect of herbivorous pests (Renault et al., 2018). Due to the presence of herbivorous pests, only invasive plants with a high level of defense ability have achieved successful invasion. This research confirms that invasive plants do have a stronger defensive ability than native plants, and also maintain a positive defensive strategy during the invasion process.

In addition to phenols, plants synthesize organic acids and their derivatives, such as jasmonic acid, hydrogen peroxide, and ethylene. These substances are usually used as signal molecules for resisting diseases and pests. After a certain part of the plant is damaged, the signal is transmitted to the entire plant and induces defenses in the undamaged part of the plant (Arce et al., 2021). Jasmonic acid and its derivative methyl jasmonate are plant growth regulators. They act as “messengers” in plant defense responses. They do not have a direct impact on normal growth and development of insects but can induce the gene expression of plant-related defenses and participate in the synthesis of defense-related substances. The content of jasmonic acid secreted by plants will increase significantly under stresses such as insect feeding or mechanical damage (Zhuang et al., 2021). The results of this study were consistent with previous studies. Compared with the level before damage, the content of jasmonic acid in invasive plants and non-invasive plants all increased significantly after the leaves were damaged. This showed that plants will have a positive signal response after being injured by insects. The injured plants can adjust resource allocation strategies and then increase their overall investment in chemical defense.

## Conclusions

The ERH is limited to situations where the invasive plant population is affected by specialist enemies. However, some of invasive plants still face the threat of generalist herbivorous insects in the invasion area, and invasive plants will maintain a high content of chemical defense capacity to reduce insect attack. The leaf hole distribution of native plants around invasive plants tested the defense of invasive plants in this study. The existence of invasive plants can change the distribution pattern of native herbivorous insects and increase their damage to surrounding native plants, favoring invasion in environments with high herbivore pressure. This possibility should be considered when assessing the stability of invasive plants in local ecosystems.

When invasive plants are damaged, they have higher induced resistance than native plants. The content of secondary metabolites related to defense increases significantly, while the content of substances related to growth decreases. Therefore, our data confirmed that invasive plants kept an active defense investment at invasion sites. In contrast to the argument that invasive plants transfer defense to growth and reproduction, invasive plants retain active defense capabilities, which can be used to explain the mechanism of plant invasion.

## Data availability statement

The raw data supporting the conclusions of this article will be made available by the authors, without undue reservation.

## Author contributions

JZ: Investigation, Methodology, Writing – original draft, Formal Analysis. BH: Investigation, Methodology, Writing – review & editing. FH: Investigation, Methodology, Writing – review & editing. GY: Investigation, Methodology, Writing – review & editing. ZL: Investigation, Methodology, Writing – review & editing. EP-Y: Writing – review & editing, Conceptualization. HX: Conceptualization, Writing – review & editing. LG: Conceptualization, Writing – review & editing, Data curation, Formal Analysis, Investigation, Methodology, Writing – original draft.

## Supplementary material

Supplementary Figure 1 Sample sites for the field investigation of the leaf hole distribution of native plant leaves around invasive plants in different vegetation types, with a map of the Guangdong Province, China. The numbers on the map are the ID numbers of each sample site, and the geographical information and common plants of each sample site are listed in **Supplementary Table S1**.

Supplementary Figure 2 The leaf holes on native plants around invasive plants were investigated, and the linear distance between each leaf hole and invasive plants was recorded.

Supplementary Figure 3 Feeding preference of two insects (*S. litura* and *S. frugiperda*) toward invasive and native species. Invasive plants include *S. trilobata*, *M. micrantha*, and *I. cairica*. Native plants include *S. calendulacea*, *I. batatas*, and *P. frutescens.* The two photos on the top show the leaves of invasive plant and native plant before and after the feeding preference experiment with *S. litura* or *S. frugiperda*. The following photos show leaf herbivory by *S. litura* or *S. frugiperda*.
