# Supplementary figures and images for: Active defense strategies for invasive plants may alter the distribution pattern of pests in the invaded area
Source: Front Plant Sci. 2024 Jul 11;15:1428752. doi: 10.3389/fpls.2024.1428752 (PMC11269258; doi:10.3389/fpls.2024.1428752)

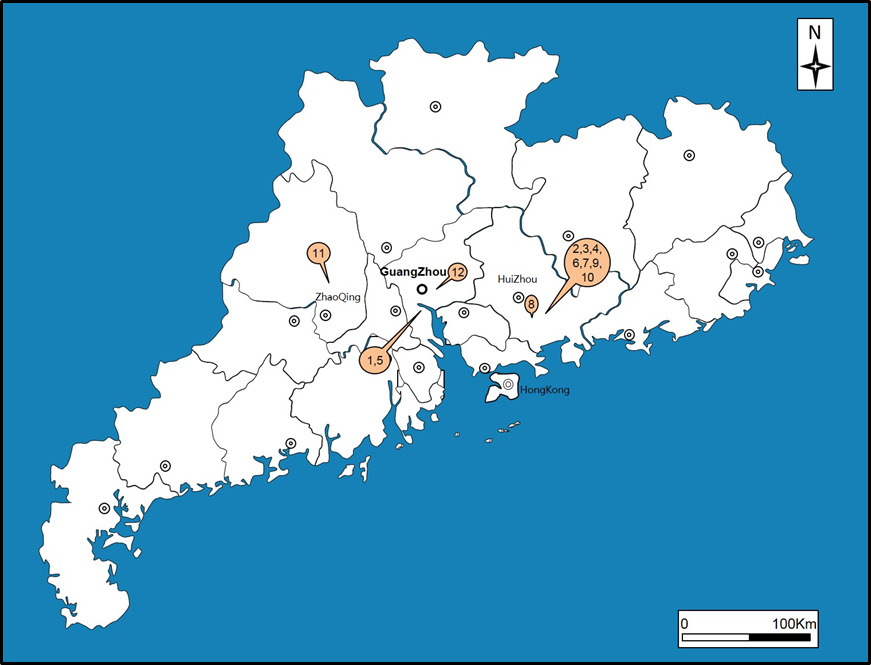

Supplement: Supplementary Figure 1 — Sample sites for the field investigation of the leaf hole distribution of native plant leaves around invasive plants in different vegetation types, with a map of the Guangdong Province, China. The numbers on the map are the ID numbers of each sample site, and the geographical information and common plants of each sample site are listed in Supplementary Table S1 . [file Image_1.tif]

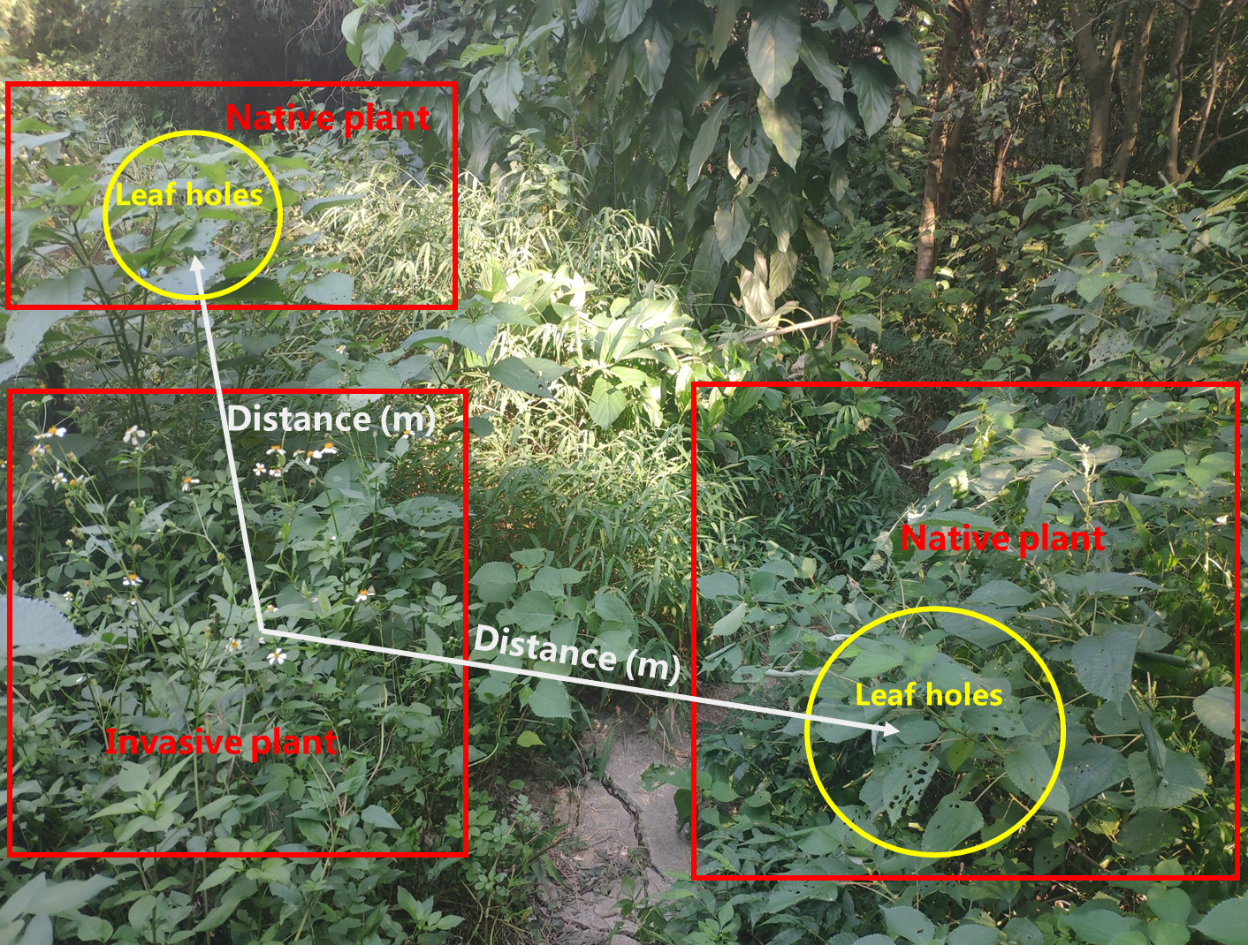

Supplement: Supplementary Figure 2 — The leaf holes on native plants around invasive plants were investigated, and the linear distance between each leaf hole and invasive plants was recorded. [file Image_2.tif]

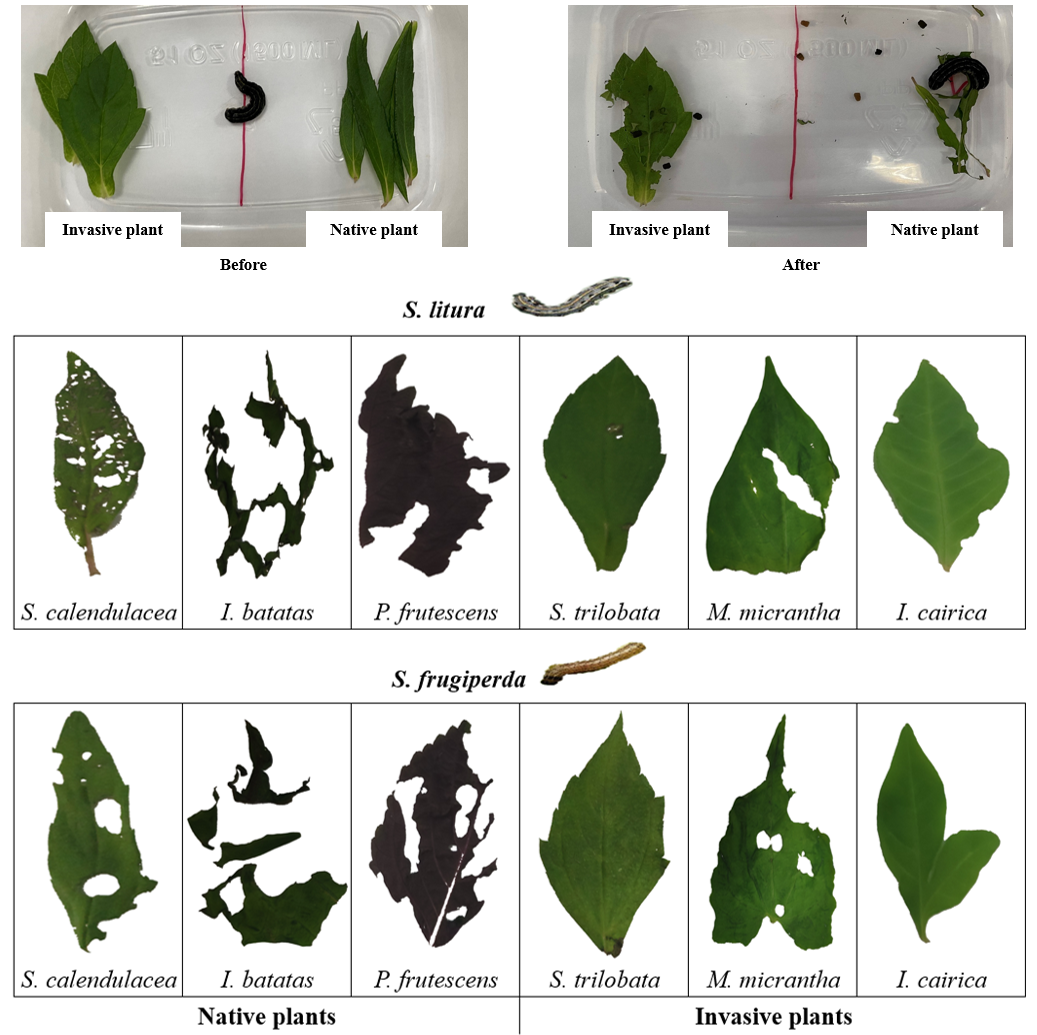

Supplement: Supplementary Figure 3 — Feeding preference of two insects (S. litura and S. frugiperda) toward invasive and native species. Invasive plants include S. trilobata, M. micrantha, and I. cairica. Native plants include S. calendulacea, I. batatas, and P. frutescens. The two photos on the top show the leaves of invasive plant and native plant before and after the feeding preference experiment with S. litura or S. frugiperda. The following photos show leaf herbivory by S. litura or S. frugiperda. [file Image_3.tif]
