# Supplementary material for: Active defense strategies for invasive plants may alter the distribution pattern of pests in the invaded area
Source: Front Plant Sci. 2024 Jul 11;15:1428752. doi: 10.3389/fpls.2024.1428752 (PMC11269258; doi:10.3389/fpls.2024.1428752)
Supplement: Supplementary file 4 [file Table_1.docx]

**Table S1.** Sites of the field investigation of the leaf holes on leaves fed on by native insects under the effects of invasive plants. PY (Panyu District of Guangzhou), GZ (Guangzhou City), HY (Huiyang District of Huizhou), HZ (Huizhou city), DH (Dinghu District of Zhaoqing), ZQ (Zhaoqing city), TH (Tianhe District of GZ), ID. refers to the number of each sample site on the map (Supp. Fig. S1)

| **Types** | **ID.** | **Site** | **Latitude and Longitude** | **Invasive Plants** | **Native Plants** |
| --- | --- | --- | --- | --- | --- |
| Orchard | 1 | Bio-island, PY, GZ | N23°3′9.61″  E113°23′3.66″ | *Bidens pilosa* L.  *Mikania micrantha* Kunth,  *Sphagneticola trilobata* (L.) Pruski | *Clausena lansium* (Lour.) Skeels  *Litchi chinensis* Sonn.  *Ipomoea nil* (L.) Roth  *Alocasia macrorrhiza* (L.) Schott  *Bauhinia purpurea* L.  *Polygonum hydropiper* L.  *Ipomoea batatas* (L.) Lam.  *Perilla frutescens* (L.) Britt. |
|  | 2 | Huaguo village, HY, HZ | N22°52′22.52″  E114°21′21.36″ | *M. micrantha*  *S. trilobata* | *L. chinensis*  *Maesa japonica* (Thunb.) Moritzi  *A. macrorrhizat* |
|  | 3 | Shanding village, HY, HZ | N22°55′49.9″  E114°20′11.6″ | *M. micrantha*  *B. pilosa* | *L. chinensis*  *M. japonica*  *A. macrorrhiza* |
|  | 4 | Daguang village, HY, HZ | N22°93'77.82''  E114°30'39.93'' | *M. micrantha* | *L. chinensis*  *Annona squamosa* L.  *Dimocarpus longan* Lour. |
| Farmland | 5 | Biological island, PY, GZ | N23°3′9.61″  E113°23′3.66″ | *B. pilosa*  *S. trilobata* | *Ricinus communis* L.  *P. frutescens*  *Hibiscus tiliaceus* L.  *B. purpurea* |
|  | 6 | Xintang village, HY, HZ | N23°1′15″  E114°24′13″ | *B. pilosa*  *M. micrantha* | *Raphanus sativus*  *Brassica juncea* (L.) Czern.et Coss  *Brassica oleracea* var.capitata L.  *Ipomoea aquatica* Forsk  *Boehmeria nivea* (L.) Gaudich |
|  | 7 | Huaguo village, HY, HZ | N22°52′22.52″  E114°21′21.36″ | *M. micrantha* | *R. sativus* |
| Wasteland | 8 | Huizhou University, HY, HZ | N23°2′23.5″  E114°24′48.28″ | *B. pilosa*  *S. trilobata* | *Hibiscus rosa-sinensis* L.  *Lagerstroemia speciosa* (L.) Pers.  *Cinnamomum camphora* (L.) Presl  *B. purpurea*  *Hibiscus tiliaceus* L. |
|  | 9 | Xintang village, HR, HZ | N23°1′15″  E114°24′13″ | *B. pilosa* | *B. nivea* |
|  | 10 | Huaguo village, HY, HZ | N22°52′22.52″  E114°21′21.36″ | *M. micrantha* | *B. nivea*  *Ficus religiosa* L. |
| Forest | 11 | Dinghushan National Forest Park, DH, ZQ | N23°10′22.12″  E112°32′25.04″ | *B. pilosa*  *S. trilobata*  *M. micrantha* | *B. nivea*  *Sterculia nobilis* Smith  *Caryota maxima* Blume ex Martius  *Mallotus paniculatus* (Lam.) Muell. Arg.  *Maesa perlarius* (Lour.) Merr. |
|  | 12 | Huolushan Forest Park, TH, GZ | N23°11'14.57''  E113°23'14.14'' | *M. micrantha*  *S. trilobata*  *B. pilosa* | *B. purpurea*  *Acacia mangium* Willd.  *Alchornea rewioides*  *Psychotria rubra* (Lour.) Poir. |
